# Genetic Associations of Parkinson’s Disease Clinical, Pathological, and Data-Driven Subtypes

**DOI:** 10.3390/genes17040449

**Published:** 2026-04-13

**Authors:** Ahmed Negida, Moaz Elsayed Abouelmagd, Belal Mohamed Hamed, Yousef Hawas, Aya Dziri, Yasmin Negida, Brian D. Berman, Matthew J. Barrett

**Affiliations:** 1 Neurology Department, Virginia Commonwealth University, Richmond, VA 23298, USA; yasmin.negeda@vcuhealth.org (Y.N.); brian.berman@vcuhealth.org (B.D.B.); matthew.barrett@vcuhealth.org (M.J.B.); 2 Faculty of Medicine, Cairo University, Cairo 11956, Egypt; moaz.s.sayed@gmail.com; 3 Faculty of Medicine, Al-Azhar University, Cairo 11884, Egypt; bm030637@gmail.com; 4 Faculty of Medicine, Tanta University, Tanta 31527, Egypt; yousef.ahmed.hawas7@gmail.com; 5 Faculty of General Medicine, Dnipro State Medical University, 49044 Dnipro, Ukraine; draya2307@gmail.com

**Keywords:** Parkinson’s disease, subtypes, genetics, *LRRK2*, *GBA1*, *SNCA*, *APOE*, alpha-synuclein, seed amplification assay, brain-first, body-first, diffuse malignant

## Abstract

**Background:** Parkinson’s disease (PD) is clinically heterogeneous, yet the genetic architecture underlying this heterogeneity remains incompletely understood. We examined the genetic correlates of four complementary PD subtyping frameworks: the clinical motor subtype (tremor-dominant [TD] vs. postural instability/gait difficulty [PIGD]), alpha-synuclein seed amplification assay status (SAA+ vs. SAA−), the pathological subtype (brain-first vs. body-first, based on the presence of REM sleep behavior disorder), and the data-driven subtype (diffuse malignant [DM] vs. mild-motor predominant [MMP] vs. intermediate [IM]). **Methods:** We analyzed 1390 PD patients from the Parkinson’s Progression Markers Initiative (PPMI) with genotypes available for seven PD-associated genes (*LRRK2*, *GBA1*, *SNCA*, *PRKN*, *PINK1*, *PARK7*, *VPS35*), including specific variant resolutions (*LRRK2 G2019S*, *R1441G/C/H*; *GBA1 N409S*, severe variants; *SNCA* *A53T*), and *APOE* (ε2/ε3/ε4 alleles). Genetic variant frequencies were compared across subtypes using chi-square or Fisher’s exact tests with the Benjamini–Hochberg false discovery rate (FDR) correction. Effect sizes were quantified using Cramér’s V. multivariable logistic regression estimated adjusted odds ratios with Wald-based 95% confidence intervals. **Results:** Among genotyped PD patients, *LRRK2* carriers constituted 13.7% (190/1390; 170 *G2019S*, 18 *R1441G/C/H*), *GBA1* 8.6% (119/1390; 96 *N409S*, 23 severe), and *SNCA* 2.0% (28/1390; all *A53T*). *APOE* ε4 carriers comprised 23.4% (323/1380). SAA-negative patients were markedly enriched for *LRRK2* variants (37.1% vs. 10.2%, *p* = 3.7 × 10^−19^, q < 0.001, V = 0.25), specifically *G2019S* (28.5% vs. 9.6%, *p* = 4.9 × 10^−11^, q < 0.001) and *R1441G/C/H* (7.9% vs. 0.5%, *p* = 2.7 × 10^−12^, q < 0.001). Body-first PD was enriched for *GBA1* carriers (12.3% vs. 6.7%, *p* = 0.004, q = 0.021) and had less *LRRK2* carriers (7.9% vs. 15.0%, *p* = 0.002, q = 0.013). The DM subtype had the highest *GBA1* frequency (14.0% vs. MMP 5.9%, *p* < 0.001, q = 0.003). After FDR correction, 10 out of 48 univariate tests remained significant. Clinical subtypes (TD vs. PIGD) showed only nominal *LRRK2* differences that did not survive FDR correction. The *APOE* genotype did not differ across any framework. **Conclusions:** PD subtypes defined by alpha-synuclein pathology (SAA), pathological onset pattern (brain-first/body-first), and data-driven classification (DM/MMP/IM) show distinct genetic profiles that survive multiple comparison correction. *LRRK2* variants strongly associate with SAA negativity (V = 0.25); *GBA1* variants associate with the severe body-first onset and the diffuse malignant subtype.

## 1. Introduction

Parkinson’s disease (PD) is the second most common neurodegenerative disorder, affecting over 10 million people worldwide [1,2,3]. Despite a shared core pathology of dopaminergic neuronal loss and alpha-synuclein aggregation, PD exhibits remarkable clinical heterogeneity in motor presentation, non-motor burden, and disease trajectory [4,5,6,7,8]. This heterogeneity has motivated the development of multiple subtyping frameworks, each capturing different aspects of disease biology [8,9,10,11,12,13,14,15,16,17,18,19].

Four major subtyping approaches have emerged. First, the clinical motor subtype classification distinguishes tremor-dominant (TD) from postural instability/gait difficulty (PIGD) subtypes based on the ratio of tremor to PIGD scores from the Movement Disorder Society–Unified Parkinson’s Disease Rating Scale (MDS-UPDRS), a clinician- and patient-rated scale assessing motor and non-motor aspects of PD [10,20]. Second, the alpha-synuclein seed amplification assay (SAA) status dichotomizes patients based on cerebrospinal fluid (CSF) alpha-synuclein seeding activity, a laboratory technique that detects misfolded alpha-synuclein aggregates—the hallmark protein of Lewy body pathology—with approximately 5–10% of clinically diagnosed PD patients testing SAA negative [21,22,23]. Third, the pathological subtype classification, proposed by Borghammer [18,19], uses baseline REM sleep behavior disorder (RBD) severity—a parasomnia in which patients physically enact dreams during sleep—to infer whether PD pathology originated in the brainstem (brain-first) or the peripheral autonomic nervous system (body-first). Fourth, the data-driven subtype classification of Fereshtehnejad et al. [15,16] integrates motor and non-motor measures into a data-driven taxonomy of diffuse malignant (DM), mild-motor predominant (MMP), and intermediate (IM) subtypes.

The genetic architecture of PD is increasingly well characterized [reviewed in 5–7]. Pathogenic variants in *LRRK2* (most commonly *G2019S* and *R1441G/C/H*) are established monogenic causes of PD with variable, often reduced, penetrance [24,25]. Variants in *GBA1* (most commonly *N409S*, with severe variants such as L483P) represent the most common genetic risk factor for PD, increasing risk approximately 5–30-fold depending on variant severity, but are not considered monogenic causes [26,27]. Less common monogenic causes include *SNCA* (*A53T*), *PRKN*, *PINK1*, *PARK7*, and *VPS35*. Multiple studies have demonstrated that *LRRK2*-associated PD frequently lacks Lewy body pathology and may show negative alpha-synuclein SAA results [25,28,29], while *GBA1*-associated PD is characterized by a more aggressive disease with faster cognitive and motor decline [30,31]. Additionally, the *APOE* ε4 allele, a major risk factor for Alzheimer’s disease, has been investigated as a potential modifier of cognitive decline in PD [32,33,34].

Whether these genetic variants differentially distribute across PD subtypes has important implications for understanding disease mechanisms and for stratifying patients in clinical trials. However, no prior study has systematically compared the genetic architectures captured by multiple subtyping frameworks within a single cohort. In this study, we address two aims: (1) to determine which PD subtyping frameworks yield the most robust genetic associations after rigorous multiple comparison correction, and (2) to compare how clinical (TD/PIGD), biological (SAA), pathological (brain-first/body-first), and data-driven (DM/MMP/IM) classification systems capture the genetic heterogeneity of PD. We analyze data from the Parkinson’s Progression Markers Initiative (PPMI; https://www.ppmi-info.org/), a multicenter longitudinal observational study, with specific variant-level resolution for the major PD-associated genes.

## 2. Materials and Methods

### 2.1. Study Population

We analyzed data from the PPMI (https://www.ppmi-info.org/; data accessed 26 January 2026), a multicenter, longitudinal observational study. Inclusion criteria were: (1) established diagnosis of PD per PPMI enrollment criteria, (2) baseline clinical assessments available, and (3) genetic testing data available. Patients without genetic testing data (n = 207) were excluded, yielding a final analytical cohort of 1390 PD patients. The study was approved by institutional review boards at all participating sites, and all participants provided written informed consent.

### 2.2. Genetic Testing

Genetic data were derived from the Indiana University genetic consensus file (January 2026), which integrates results from CLIA-certified clinical genetic testing, genome-wide association studies (GWAS), whole-exome sequencing (WES), whole-genome sequencing (WGS), and Sanger sequencing. Variant pathogenicity was determined by the PPMI genetics core using American College of Medical Genetics and Genomics (ACMG) guidelines and ClinVar annotations. We analyzed seven PD-associated genes: *LRRK2*, *GBA1*, *SNCA*, *PRKN*, *PINK1*, *PARK7*, and *VPS35*. Binary carrier status was defined as harboring at least one pathogenic or likely pathogenic variant as classified in ClinVar.

For *LRRK2*, we separately identified the *G2019S* and *R1441G/C/H* variants. For *GBA1*, we distinguished the *N409S* (mild) variant from severe variants (L483P, IVS2 + 1G > A, 84GG, and others), consistent with the classification by Gan-Or et al. and prior PPMI publications. For *SNCA*, we identified the *A53T* variant. Carrier status includes both heterozygous and homozygous carriers. For autosomal dominant genes (*LRRK2*, *SNCA*), heterozygous carriers are considered affected; for *GBA1*, both heterozygous and homozygous carriers are considered to be at increased risk.

*APOE* genotyping was available for 1380 PD patients. ε4 carrier status (at least one ε4 allele), ε4 homozygosity (ε4/ε4), and ε2 carrier status were computed. Full genotype distributions (ε2/ε2, ε2/ε3, ε2/ε4, ε3/ε3, ε3/ε4, ε4/ε4) were tabulated across subtypes.

### 2.3. Subtype Classification

#### 2.3.1. Clinical Motor Subtypes (TD/PIGD)

Classification followed Stebbins et al. (2013) [20]. The tremor score was the mean of 10 MDS-UPDRS Part III tremor items (items 3.15a, 3.15b, 3.16a, 3.16b, 3.17a, 3.17b, 3.17c, 3.17d, 3.17e, 3.18). The PIGD score was the mean of 5 items (Part III items 3.10, 3.11, 3.12; Part II items 2.12, 2.13). A tremor/PIGD ratio ≥ 1.15 classified TD; ≤0.90 classified PIGD; intermediate ratios were classified as indeterminate [10,20].

#### 2.3.2. SAA Status (SAA+/SAA−)

Classification was based on cerebrospinal fluid (CSF) alpha-synuclein seed amplification assay results using the Amprion platform (Amprion Inc., San Diego, CA, USA), a commercially available real-time quaking-induced conversion (RT-QuIC)-based assay that detects misfolded alpha-synuclein seeds in CSF [28]. Positive (SAA+) and negative (SAA−) results were used as reported by the PPMI biomarker core.

#### 2.3.3. Pathological Subtype (Brain-First/Body-First)

Classification followed the Borghammer [18,19] model using the baseline RBD Screening Questionnaire (RBDSQ) total score, computed as the sum of 12 binary items from questions Q1–Q9 (range 0–12; Q6 contributes 4 sub-items, Q10 is excluded in PD cohorts). A score ≥ 6 classified body-first PD; ≤3 classified brain-first PD; scores of 4–5 were classified as indeterminate [18,19,35].

#### 2.3.4. Data-Driven Subtype (DM/MMP/IM)

Classification followed Fereshtehnejad et al. [15,16]. Baseline Z-scores were computed for three motor composites (PIGD/tremor ratio, MDS-UPDRS Part III OFF-medication total, MDS-UPDRS Part II total) and three non-motor measures (Montreal Cognitive Assessment [MoCA; inverted so higher = worse], RBD Screening Questionnaire [RBDSQ] total, Scales for Outcomes in Parkinson’s Disease–Autonomic [SCOPA-AUT] total). The 75th percentile of each defined the worst quartile. Patients with a motor composite Z-score ≥ 75th percentile and ≥1 non-motor Z-score ≥ 75th percentile, or with all 3 non-motor Z-scores ≥ 75th percentile, were classified as DM. Those with motor composite Z-score < 75th percentile and all non-motor Z-scores < 75th percentile were classified as MMP. All remaining patients were classified as IM.

### 2.4. Statistical Analysis

Genetic variant carrier frequencies were compared across subtypes using Pearson’s chi-squared test or Fisher’s exact test when any expected cell count was <5. All *p*-values are two-sided. To account for multiple testing across 48 univariate comparisons (12 genetic variants × 4 subtyping schemes), we applied the Benjamini–Hochberg procedure to control the false discovery rate (FDR) at 5%. FDR-adjusted *p*-values (q-values) are reported alongside nominal *p*-values [36]. Effect sizes for categorical associations were quantified using Cramér’s V [37].

Multivariable logistic regression was performed using maximum-likelihood estimation (statsmodels Logit) to estimate adjusted odds ratios (OR) with Wald-based 95% confidence intervals (CI) and *p*-values. Models included age at baseline visit (standardized), *LRRK2* carrier status (heterozygous or homozygous for any pathogenic variant), *GBA1* carrier status (heterozygous or homozygous), *APOE* ε4 carrier status (at least one ε4 allele), and sex as covariates. *APOE* ε2 carrier status was not included as a separate covariate given the absence of an a priori hypothesis and to limit the number of predictors relative to events. Model fit was assessed using McFadden’s pseudo-R^2^ and Akaike information criterion (AIC). For predictors exhibiting perfect or quasi-complete separation, results are reported as not estimable.

*APOE* genotype distributions were compared across subtypes using chi-square tests on the full 6-level genotype table. Cross-scheme agreement between subtyping frameworks was assessed using Cramér’s V on cross-tabulations of subtype assignments. All analyses were performed in Python 3.13 using pandas, scipy, statsmodels, and matplotlib. Code and data processing pipelines are available upon request. Missing data were handled using a complete-case (available-case) analysis strategy, where each subtyping framework analysis included all patients with both genetic data and the relevant classification variable. Genetic data were available for 1390/1597 enrolled patients (87.0%); SAA results for 1268 (79.4%); RBDSQ scores for 1560 (97.7%); and complete data for data-driven classification for 1272 (79.6%). For the multivariable logistic regression models, which required non-missing values for all covariates, effective sample sizes ranged from 560 to 600. This complete-case approach assumes data are missing at random (MAR).

## 3. Results

### 3.1. Study Cohort

The analytical cohort comprised 1390 PD patients with genetic testing data (mean age 63.1 ± 9.8 years, 65.6% male, Table 1). Of these, 190 (13.7%) carried *LRRK2* variants (170 *G2019S*, 18 *R1441G/C/H*, 2 other; all heterozygous), 119 (8.6%) carried *GBA1* variants (96 *N409S*, 23 severe; all heterozygous except 2 homozygous), 28 (2.0%) carried *SNCA* *A53T*, and 17 (1.2%) carried *PRKN* variants. No patients carried pathogenic or likely pathogenic *PINK1*, *PARK7*, or *VPS35* variants. Overall, 287 patients (20.6%) carried at least one pathogenic variant.

*APOE* genotyping was available for 1380 PD patients. *APOE* ε4 carriers comprised 23.4% (323/1380), including 24 ε4/ε4 homozygotes (1.7%). *APOE* ε2 carriers comprised 15.1% (209/1380).

### 3.2. Subtype Distributions

- Clinical (TD/PIGD): At the baseline visit, among 1220 patients with evaluable tremor/PIGD scores, 793 (65.0%) were classified as TD, 296 (24.3%) as PIGD, and 131 (10.7%) as indeterminate.
- SAA Status: Among 1268 patients with baseline SAA results, 1112 (87.7%) were SAA+ and 156 (12.3%) were SAA−.
- Pathological Subtype: Among 1560 patients with baseline RBDSQ data, 985 (63.1%) were classified as brain-first, 342 (21.9%) as body-first, and 233 (14.9%) as indeterminate.
- Data-Driven Subtype: Among 1272 patients with complete baseline data, 322 (25.3%) were classified as DM, 441 (34.7%) as MMP, and 509 (40.0%) as IM.

The four subtyping frameworks showed low inter-scheme agreement (Supplementary Materials, Figure S2), with the highest concordance between data-driven and pathological (Cramér’s V = 0.29) and data-driven and clinical (V = 0.25) classifications. This low inter-scheme agreement is consistent with prior reports showing that different PD subtyping methods capture largely non-overlapping aspects of disease heterogeneity [12,38].

### 3.3. Genetic Correlates by Subtyping Framework

#### 3.3.1. Clinical Motor Subtype (TD vs. PIGD)

**Figure 1:**
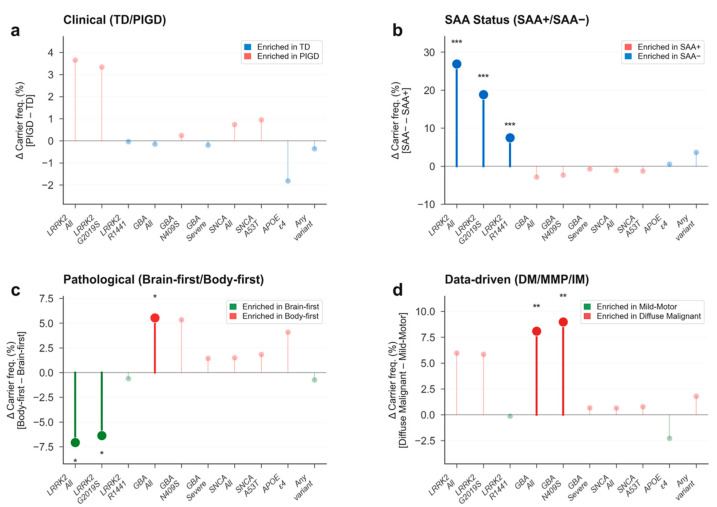
Diverging carrier frequency differences between subtypes. Vertical diverging lollipop plots showing the difference in carrier frequency (%) between subtype groups. (**a**) Clinical (PIGD − TD). (**b**) SAA (SAA− − SAA+). (**c**) Pathological (body-first − brain-first). (**d**) Data-driven (DM − MMP). Stars indicate FDR significance (q < 0.05). TD, tremor-dominant; PIGD, postural instability/gait difficulty; SAA, seed amplification assay; DM, diffuse malignant; MMP, mild-motor predominant; FDR, false discovery rate. * = *p* < 0.05; ** = *p* < 0.01; *** = *p* < 0.001.

Genetic variant frequencies showed only marginal differences between TD and PIGD subtypes (Table 2, Figure 1a). *LRRK2* carrier frequency was higher in PIGD (7.0% 19/270) than TD (3.4% [25/739], *p* = 0.024, q = 0.095, V = 0.07), but this did not survive FDR correction. No significant differences were observed for *GBA1* (PIGD 3.0% vs. TD 3.1%, *p* = 1.0), *SNCA* (PIGD 0.9% vs. TD 0.0%, *p* = 0.07), *PRKN*, *APOE* ε4 (PIGD 22.7% vs. TD 24.5%, *p* = 0.54), or any other tested variant. *APOE* genotype distribution did not differ between TD and PIGD (χ^2^ = 4.28, df = 5, *p* = 0.51).

In adjusted logistic regression (n = 586; reduced from univariate sample due to complete-case analysis; pseudo-R^2^ = 0.014, AIC = 669.5), no predictor reached significance for PIGD classification: *LRRK2* (OR = 2.41 [0.63–9.17], *p* = 0.20), *GBA1* (not estimable due to separation), *APOE* ε4 (OR = 0.76 [0.48–1.21], *p* = 0.24), age (OR = 0.97 [0.80–1.16], *p* = 0.72), male sex (OR = 1.26 [0.84–1.88], *p* = 0.26).

#### 3.3.2. SAA Status (SAA+ vs. SAA−)

**Figure 2:**
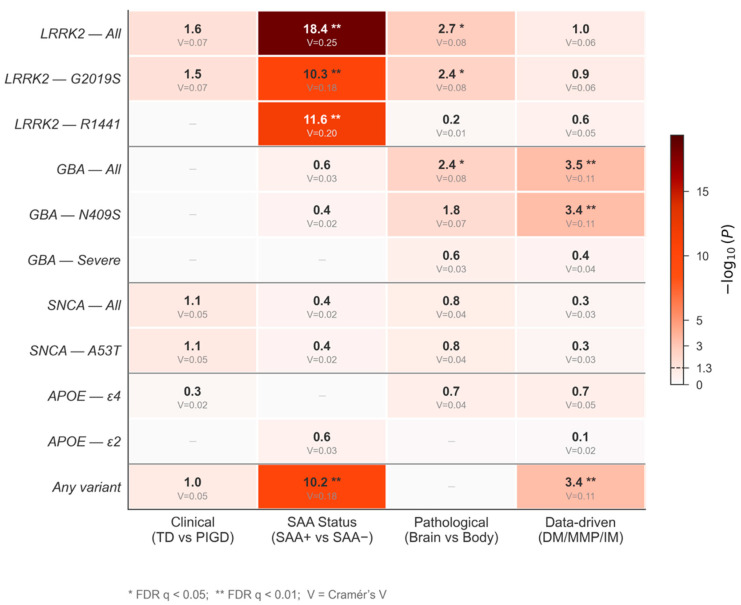
Genetic–subtype association heatmap. Color intensity represents −log_10_(P); Cramér’s V effect sizes are annotated within cells. Asterisks indicate FDR-significant associations (q < 0.05). TD, tremor-dominant; PIGD, postural instability/gait difficulty; SAA, seed amplification assay; DM, diffuse malignant; MMP, mild-motor predominant; IM, intermediate; FDR, false discovery rate.

SAA-negative patients showed dramatically higher rates of *LRRK2* variants (Table 3, Figure 1b). Overall *LRRK2* carrier frequency was 37.1% in SAA− vs. 10.2% in SAA+ (*p* = 3.7 × 10^−19^, q < 0.001, V = 0.25). This was driven by both *G2019S* (28.5% vs. 9.6%, *p* = 4.9 × 10^−11^, q < 0.001, V = 0.18) and *R1441G/C/H* (7.9% vs. 0.5%, *p* = 2.7 × 10^−12^, q < 0.001, V = 0.20). Any pathogenic variant carrier status was significantly higher in SAA− (43.0% vs. 19.2%, *p* = 6.4 × 10^−11^, q < 0.001, V = 0.18, Figure 2). *GBA1* did not differ between SAA groups (SAA− 4.6% vs. SAA+ 7.5%, *p* = 0.28). Neither *APOE* ε4 (*p* = 0.89) nor *APOE* genotype distribution (χ^2^ = 6.34, df = 5, *p* = 0.27) differed between SAA groups.

In adjusted logistic regression (n = 600, complete-case analysis; pseudo-R^2^ = 0.018, AIC = 440.3), *LRRK2* carrier status was the only significant predictor of SAA+ status (OR = 0.22 [0.06–0.78], *p* = 0.02), reflecting the strong enrichment of *LRRK2* variants in the SAA-negative group. *GBA1* was not estimable due to quasi-complete separation.

#### 3.3.3. Pathological Subtype (Brain-First vs. Body-First)

Body-first PD was enriched for *GBA1* carriers (12.3% [37/302] vs. 6.7% [59/879] in brain-first, *p* = 0.004, q = 0.021, V = 0.08, Table 4, Figure 1c), with *GBA1* *N409S* showing a nominal enrichment (12.0% vs. 6.6%, *p* = 0.015) that did not survive FDR correction (q = 0.067). Conversely, body-first PD was depleted for *LRRK2* carriers (7.9% [24/302] vs. 15.0% [132/879], *p* = 0.002, q = 0.013, V = 0.08, Figure 2), driven by *G2019S* (7.0% vs. 13.3%, *p* = 0.004, q = 0.020, V = 0.08, Figure 2). *APOE* ε4 did not differ (body-first 26.9% vs. brain-first 22.8%, *p* = 0.20). *APOE* genotype distribution was also non-significant (χ^2^ = 7.98, df = 5, *p* = 0.16).

In adjusted logistic regression (n = 595, pseudo-R^2^ = 0.039, AIC = 671.7), male sex was the strongest predictor of body-first classification (OR = 2.14 [1.38–3.31], *p* = 6.3 × 10^−4^), followed by age (OR = 1.37 [1.13–1.67], *p* = 0.002). *LRRK2* (OR = 0.58 [0.13–2.69], *p* = 0.49), *GBA1* (OR = 1.13 [0.28–4.49], *p* = 0.86), and *APOE* ε4 (OR = 1.48 [0.96–2.27], *p* = 0.08) were non-significant.

#### 3.3.4. Data-Driven Subtype (DM vs. MMP vs. IM)

The DM subtype showed significantly elevated *GBA1* carrier frequency (14.0% [39/279] vs. IM 6.3% [28/447] vs. MMP 5.9% [24/407], *p* < 0.001, q = 0.003, V = 0.11, Table 5, Figure 1d), driven by *GBA1* *N409S* (DM 13.6% vs. MMP 4.6%, *p* < 0.001, q = 0.003, V = 0.11). Any pathogenic variant carrier status was also highest in DM (32.3% vs. IM 21.3% vs. MMP 18.2%, *p* < 0.001, q = 0.003, V = 0.11, Figure 2). *LRRK2* carrier frequency showed a non-significant trend (DM 15.1% vs. IM 12.8% vs. MMP 9.1%, *p* = 0.11, q = 0.31). *APOE* ε4 did not differ (DM 20.8% vs. IM 26.9% vs. MMP 23.1%, *p* = 0.18). *APOE* genotype was non-significant (χ^2^ = 12.94, df = 10, *p* = 0.23).

In adjusted logistic regression (n = 560, pseudo-R^2^ = 0.040, AIC = 603.7), age was the strongest predictor of DM classification (OR = 1.63 [1.31–2.03], *p* = 1.2 × 10^−5^). *LRRK2* (OR = 1.46 [0.37–5.78], *p* = 0.59), *GBA1* (OR = 1.18 [0.30–4.72], *p* = 0.81), *APOE* ε4 (OR = 0.90 [0.56–1.45], *p* = 0.66), and male sex (OR = 1.37 [0.85–2.21], *p* = 0.20) were non-significant in the multivariable model, likely due to the small number of carriers and limited power.

### 3.4. GBA1 and LRRK2 Carrier Subtype Profiles

**Figure 3:**
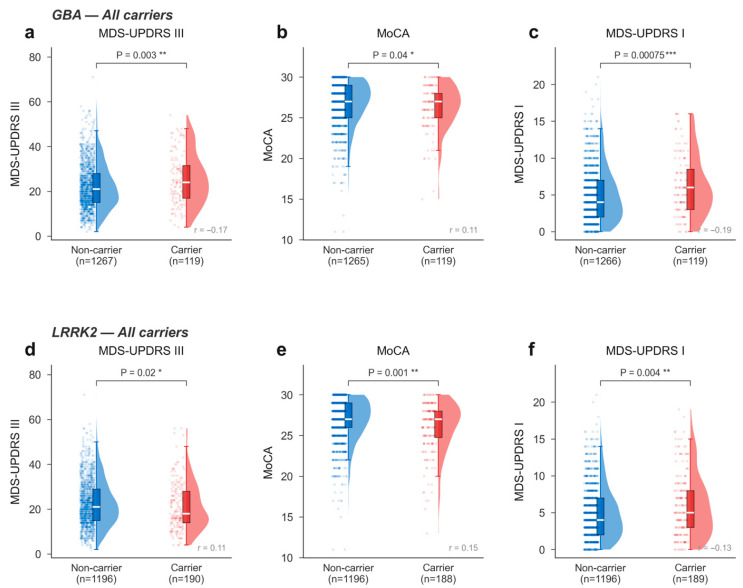
*GBA1*- and *LRRK2*-carrier clinical subtype profiles. Raincloud plots (half-violin + jitter strip + box) comparing baseline clinical measures between genetic carriers and non-carriers. (**a**–**c**) *GBA1* carriers vs. non-carriers. (**d**–**f**) *LRRK2* carriers vs. non-carriers. MDS-UPDRS, Movement Disorder Society–Unified Parkinson’s Disease Rating Scale; MoCA, Montreal Cognitive Assessment. * = *p* < 0.05; ** = *p* < 0.01; and *** = *p* < 0.001.

At the baseline visit, *GBA1* carriers showed worse baseline motor scores (MDS-UPDRS III: *p* = 0.003, rank-biserial r = −0.17, a small-to-medium effect per Cohen’s conventions where |r| = 0.10 is small, 0.30 is medium, and 0.50 is large), lower cognitive performance (MoCA: *p* = 0.04, r = −0.11), and greater non-motor burden (MDS-UPDRS I: *p* = 7.5 × 10^−4^, r = −0.19) compared with non-carriers (Figure 3a–c). *LRRK2* carriers similarly showed significant differences in motor scores (*p* = 0.02, r = 0.15), MoCA (*p* = 0.001, r = −0.15), and MDS-UPDRS I (*p* = 0.004, r = −0.13) (Figure 3d–f).

### 3.5. APOE Analysis

*APOE* ε4 carrier frequency was consistent across all four subtyping frameworks, ranging from 20.8% to 26.9% without statistically significant differences. Full *APOE* genotype distributions (ε2/ε2, ε2/ε3, ε2/ε4, ε3/ε3, ε3/ε4, ε4/ε4) were compared using chi-square tests and showed no significant associations with any subtype classification (all *p* > 0.16, Figure 2). These findings suggest that the *APOE* genotype does not meaningfully differentiate PD subtypes in this cohort. Importantly, this cross-sectional analysis does not address whether *APOE* ε4 modifies longitudinal disease progression within subtypes, which remains an important question for future studies.

## 4. Discussion

This study provides the first systematic head-to-head comparison of the genetic correlates of four complementary PD subtyping frameworks in a single well-characterized cohort (the PPMI), with specific variant-level resolution for the major PD genes and inclusion of an *APOE* genotype analysis. Our findings reveal that subtyping schemes based on biological markers (SAA status), pathological models (brain-first/body-first), and data-driven approaches (DM/MMP/IM) capture distinct genetic architectures that survive rigorous multiple comparison correction (10 of 48 univariate tests significant after FDR), whereas the traditional clinical motor subtype (TD/PIGD) does not.

The SAA-based classification yielded the most robust genetic associations. SAA-negative PD patients showed a strikingly high prevalence of *LRRK2* variants (37.1%), driven by both *G2019S* (28.5%) and *R1441G/C/H* (7.9%). The effect size was substantial (Cramér’s V = 0.25), and all SAA–*LRRK2* associations survived FDR correction with q < 0.001. This finding has a clear biological basis: *LRRK2*-PD frequently lacks Lewy body pathology at autopsy, and alpha-synuclein SAA detects the misfolded alpha-synuclein seeds that are the molecular correlate of Lewy bodies. Thus, *LRRK2* carriers who test SAA-negative likely represent individuals whose neurodegeneration proceeds through non-synuclein mechanisms—such as *LRRK2* kinase-mediated neuroinflammation, Rab GTPase dysregulation, and lysosomal dysfunction—rather than classical alpha-synuclein aggregation [24,25,39]. Autopsy studies have established that an appreciable subset of *LRRK2*-PD patients lack Lewy body pathology despite dopaminergic neuron loss and instead exhibit alternative proteinopathies such as tau or TDP-43 aggregation [25]. In the PPMI cohort, Siderowf et al. [28] reported that only 68% of *LRRK2*-PD participants were SAA-positive, mirroring the frequency of typical Lewy pathology observed at autopsy, while 96% of *GBA1*-PD and 93% of sporadic PD cases tested positive. Chahine et al. [29] further demonstrate that approximately one-third of *LRRK2* parkinsonism cases had no in vivo evidence of alpha-synuclein aggregates, contrasting sharply with only 7–9% SAA negativity in sporadic PD.

The very high enrichment of *R1441G/C/H* in SAA− (7.9% vs. 0.5%, *p* = 2.7 × 10^−12^, q < 0.001) suggests these rarer variants may have an even stronger association with SAA negativity than *G2019S*. In adjusted analysis, an *LRRK2* carrier status was the only significant predictor of SAA status (OR = 0.22 [0.06–0.78], *p* = 0.02). This differential effect by variant type aligns with reports that *R1441G/C/H* carriers present a more homogeneous subtype with higher rates of preserved olfaction and potentially lower alpha-synuclein burden than *G2019S* carriers [40].

*GBA1* variants, predominantly *N409S*, were enriched in body-first PD (12.3% vs. 6.7%, q = 0.021) and DM (14.0% vs. MMP 5.9%, q = 0.003). This aligns with the known association of *GBA1* with more aggressive disease, including faster cognitive decline, earlier autonomic dysfunction, and greater non-motor burden [26,27,30,31,41]. Indeed, *GBA1* carriers in our cohort showed worse baseline motor scores, cognitive performance, and non-motor burden (Figure 3). The enrichment of *GBA1* variants in both the body-first and DM subtypes—which share features of widespread non-motor involvement—suggests these classification systems capture overlapping aspects of *GBA1*-driven pathophysiology.

Body-first PD, characterized by prominent RBD and autonomic dysfunction, may represent a subtype where peripheral alpha-synuclein spread predominates. *GBA1* mutations reduce glucocerebrosidase activity, leading to glucosylceramide and glucosylsphingosine accumulation that directly promotes alpha-synuclein aggregation and impairs autophagic–lysosomal clearance [42,43]. This lysosomal dysfunction may be particularly consequential in peripheral autonomic neurons, where impaired degradation could facilitate earlier and more widespread synuclein propagation through the gut–brain axis, providing a mechanistic link between *GBA1* carrier status and the body-first phenotype [43,44,45].

*LRRK2* variants showed a reciprocal pattern to *GBA1*: enrichment in brain-first (15.0% vs. 7.9% body-first, q = 0.013) and SAA-negative (37.1% vs. 10.2%, q < 0.001) PD. This convergence is biologically coherent: *LRRK2*-PD frequently lacks Lewy body pathology at autopsy [25], and SAA detects the misfolded alpha-synuclein seeds that constitute Lewy pathology. *LRRK2* carriers who are both SAA-negative and brain-first likely represent individuals with predominantly nigral-striatal neurodegeneration driven by kinase-mediated mechanisms rather than classical synucleinopathy [29,39].

The TD/PIGD classification showed only nominal *LRRK2* enrichment in PIGD (7.0% vs. 3.4%, *p* = 0.024), but this did not survive FDR correction (q = 0.095). No other genetic markers differentiated these subtypes. The low cross-scheme agreement between clinical and other frameworks (V = 0.05–0.25; Supplementary Materials, Figure S2) suggests that a clinical motor subtype may not cleanly map onto distinct genetic etiologies. This is consistent with prior work showing that the TD/PIGD classification is unstable over time, with a substantial proportion of patients switching subtypes during longitudinal follow-up, suggesting it may capture a disease-stage-dependent dimension rather than a fixed biological entity [11]. Dulski et al. [5] similarly found only modest genome-wide associations with clinical motor subtypes compared with more robust genetic signals for non-motor-based classifications.

*APOE* ε4 carrier frequency and full genotype distributions were remarkably consistent across all four frameworks. While *APOE* ε4 is a major risk factor for Alzheimer’s disease, our findings do not support its role in defining PD subtypes. This is consistent with prior studies showing that *APOE* ε4’s role in PD is primarily in modifying cognitive outcomes rather than defining motor or pathological subtypes. Multiple studies have linked *APOE* ε4 to faster cognitive decline in PD [32,33], while associations with motor severity and motor subtypes have been largely inconclusive [38,46,47,48,49]. A recent PPMI-based longitudinal study confirmed that *APOE* ε4 accelerates cognitive decline specifically in sporadic PD but not in *GBA1*-PD or *LRRK2*-PD, underscoring the subtype-specific effects of *APOE* on cognition rather than motor subtype [34]. Taken together, these findings confirm that *APOE* is a modifier of cognitive trajectory rather than a determinant of PD subtype membership. Critically, our cross-sectional analysis does not rule out the possibility that *APOE* ε4 carriers within a given subtype may exhibit faster cognitive decline or different progression trajectories than non-carriers in the same subtype. Longitudinal studies examining *APOE* as a modifier of within-subtype progression remain an important future direction.

The four subtyping frameworks showed generally low concordance, indicating they capture different aspects of PD biology. The highest agreement was between data-driven and pathological subtypes (V = 0.29), consistent with the overlap in non-motor features (particularly RBD) used by both approaches. The clinical framework showed particularly low agreement with all others (V = 0.05–0.25), reinforcing that motor subtype classification captures a distinct—and genetically less informative—dimension of heterogeneity. This low concordance between subtyping frameworks has been observed previously by Chen et al. [12], who compared multiple data-driven PD subtyping methods and found that agreement was highly dependent on the clinical domains incorporated. Our genetic data provide a complementary perspective: subtyping frameworks that incorporate non-motor features and biological markers (SAA, RBD, multi-domain composites) capture more genetically informative dimensions of PD heterogeneity than purely motor-based classifications.

Our findings have implications for clinical trial design. SAA status should be considered as a stratification variable for *LRRK2*-targeted trials (e.g., kinase inhibitors), given the strong association with *LRRK2* carrier status (V = 0.25). In our data, 37.1% of SAA-negative patients carry *LRRK2* variants, but 62.9% do not—meaning SAA status alone is an imperfect proxy for the genotype. Conversely, 69.5% of *LRRK2* carriers are SAA-positive, so excluding SAA-negative patients from a *LRRK2* inhibitor trial would eliminate approximately 30% of the target population. For *GBA1*-directed therapies, body-first and DM subtypes show the highest *GBA1* carrier frequencies (12.3% and 14.0%), but since the majority of *GBA1* carriers fall outside these subtypes, genotype-first enrollment remains more efficient than phenotype-first approaches. These considerations support a combined genotype-plus-biomarker strategy for trial enrollment. The low concordance between subtyping frameworks further supports multi-dimensional stratification in adaptive platform trials [50].

This study has several limitations. First, the PPMI is an enrichment cohort with overrepresentation of genetic PD relative to the general PD population, which may inflate carrier frequencies. Second, the cross-sectional, baseline-visit design limits inference about temporal relationships between genetic status and subtype evolution. Third, the absence of *PINK1*, *PARK7*, and *VPS35* carriers reflects the extreme rarity of these variants in unselected PD cohorts and does not constitute evidence against their relevance to PD subtypes; much larger cohorts would be required. Fourth, the multivariable regression models were limited by reduced sample sizes (N = 560–600) due to complete-case analysis and should be interpreted as exploratory. Fifth, ancestry principal components were not included in regression models; while the PPMI cohort is predominantly of European ancestry (>90%), limiting population stratification bias, future analyses incorporating ancestry-informative markers would further strengthen these findings. Sixth, indeterminate patients (10.7% for clinical, 14.9% for pathological subtyping) were excluded; their genetic profiles were generally intermediate between defined subtypes without a distinctive genetic signature. Seventh, the pathological subtype uses RBDSQ as a proxy for RBD, with imperfect sensitivity and specificity for polysomnography-confirmed RBD. Finally, the complete-case approach assumes data are missing at random.

## 5. Conclusions

This study demonstrates that PD subtyping frameworks based on alpha-synuclein biology (SAA status), pathological onset pattern (brain-first/body-first), and data-driven phenotyping capture distinct genetic architectures, whereas the traditional clinical motor subtype (TD/PIGD) does not. Two key biological insights emerge. First, *LRRK2* variants—particularly *R1441G/C/H*—are markedly enriched in SAA-negative and brain-first PD, reinforcing the concept that *LRRK2*-associated neurodegeneration frequently proceeds through non-synuclein pathways, with direct implications for *LRRK2*-targeted clinical trials where SAA stratification will be essential. Second, *GBA1* variants concentrate in the more aggressive body-first and diffuse malignant subtypes, consistent with the role of lysosomal dysfunction in promoting widespread alpha-synuclein propagation. Together, these findings argue that biologically grounded classification systems should supplement traditional motor subtyping for genetic stratification in clinical trials and precision medicine approaches to PD.

## Figures and Tables

**Table 1:** Baseline demographics and clinical characteristics of the study population categorized into subtypes.

	Clinical Motor Subtypes		SAA Status		Pathological Subtypes		Data-Driven Subtypes		
	PIGD (n = 296)	TD (n = 793)	SAA+ (n = 1112)	SAA− (n = 156)	Body-First (n = 342)	Brain-First (n = 985)	DM (n = 322)	IM (n = 509)	MMP (n = 441)
Age, years	63.2 ± 9.5	63.5 ± 9.5	62.6 ± 9.6	66.1 ± 9.6	63.3 ± 9.5	62.6 ± 9.9	64.0 ± 10.3	62.7 ± 9.1	59.8 ± 10.1
Male sex, n (%)	117 (40%)	317 (40%)	377 (34%)	49 (31.4%)	148 (43%)	338 (34%)	133 (41%)	208 (41%)	160 (36%)
Education, years	15.9 ± 3.4	16.2 ± 3.0	16.2 ± 3.2	15.1 ± 4.2	15.7 ± 3.4	16.1 ± 3.4	15.9 ± 3.6	16.1 ± 3.3	16.2 ± 3.2
MDS-UPDRS III	21.6 ± 9.9	22.6 ± 9.7	22.7 ± 9.9	20.6 ± 9.0	23.7 ± 11.4	21.8 ± 10.0	28.0 ± 12.1	21.3 ± 9.6	19.5 ± 8.3
MoCA	26.9 ± 2.4	26.9 ± 2.5	26.9 ± 2.5	26.0 ± 2.8	26.4 ± 2.9	26.8 ± 2.7	25.8 ± 2.9	26.3 ± 2.6	28.1 ± 1.3
H&Y stage	2 [1,2]	2 [1,2]	2 [1,2]	2 [1,2]	2 [1,2]	2 [1,2]	2 [2]	2 [1,2]	2 [1,2]
SAA positive, n (%)	207 (83%)	647 (91%)	1112 (100%)	0 (0%)	240 (89%)	704 (86%)	218 (87%)	370 (88%)	351 (91%)
*LRRK2* carrier, n (%)	19 (7.0%)	25 (3.4%)	109 (10.2%)	56 (37.1%)	24 (7.9%)	132 (15.0%)	42 (15.1%)	57 (12.8%)	37 (9.1%)
*GBA* carrier, n (%)	8 (3.0%)	23 (3.1%)	80 (7.5%)	7 (4.6%)	37 (12.3%)	59 (6.7%)	39 (14.0%)	28 (6.3%)	24 (5.9%)
*SNCA* carrier, n (%)	2 (0.7%)	0 (0.0%)	12 (1.1%)	0 (0.0%)	9 (3.0%)	13 (1.5%)	8 (2.9%)	7 (1.6%)	9 (2.2%)
*PRKN* carrier, n (%)	3 (1.1%)	12 (1.6%)	11 (1.0%)	3 (2.0%)	4 (1.3%)	10 (1.1%)	4 (1.4%)	4 (0.9%)	6 (1.5%)
*PINK1* carrier, n (%)	0 (0.0%)	0 (0.0%)	0 (0.0%)	0 (0.0%)	0 (0.0%)	0 (0.0%)	0 (0.0%)	0 (0.0%)	0 (0.0%)
*PARK7* carrier, n (%)	0 (0.0%)	0 (0.0%)	0 (0.0%)	0 (0.0%)	0 (0.0%)	0 (0.0%)	0 (0.0%)	0 (0.0%)	0 (0.0%)
*VPS35* carrier, n (%)	0 (0.0%)	0 (0.0%)	0 (0.0%)	0 (0.0%)	0 (0.0%)	0 (0.0%)	0 (0.0%)	0 (0.0%)	0 (0.0%)
*LRRK2*-*G2019S*, n (%)	17 (6.3%)	22 (3.0%)	103 (9.6%)	43 (28.5%)	21 (7.0%)	117 (13.3%)	40 (14.4%)	51 (11.4%)	35 (8.6%)
*LRRK2*-*R1441G/C/H*, n (%)	1 (0.4%)	3 (0.4%)	5 (0.5%)	12 (7.9%)	3 (1.0%)	14 (1.6%)	1 (0.4%)	6 (1.3%)	2 (0.5%)
*GBA*-*N409S*, n (%)	6 (2.8%)	16 (2.6%)	66 (7.2%)	6 (4.8%)	30 (12.0%)	49 (6.6%)	32 (13.6%)	23 (6.2%)	16 (4.6%)
*GBA* severe, n (%)	2 (0.9%)	7 (1.1%)	14 (1.5%)	1 (0.8%)	7 (2.8%)	10 (1.3%)	7 (3.0%)	5 (1.3%)	8 (2.3%)
*SNCA*-*A53T*, n (%)	2 (0.9%)	0 (0.0%)	12 (1.3%)	0 (0.0%)	9 (3.6%)	13 (1.8%)	8 (3.4%)	7 (1.9%)	9 (2.6%)
*APOE* ε4 carrier, n (%)	61 (22.7%)	179 (24.5%)	254 (24.0%)	37 (24.5%)	81 (26.9%)	199 (22.8%)	58 (20.8%)	119 (26.9%)	93 (23.1%)
*APOE* ε2 carrier, n (%)	41 (15.2%)	114 (15.6%)	151 (14.3%)	27 (17.9%)	43 (14.3%)	131 (15.0%)	41 (14.7%)	69 (15.6%)	52 (12.9%)

Values are mean ± SD, median [IQR], or n (%). TD, tremor-dominant; PIGD, postural instability/gait difficulty; SAA, alpha-synuclein seed amplification assay; pathological subtype based on Borghammer [18,19] model using RBDSQ total score (12 items, Q1–Q9): ≥6 = body-first; ≤3 = brain-first. Indeterminate patients (n = 233) excluded; DM, diffuse malignant; IM, intermediate; MMP, mild-motor predominant. Classification per Fereshtehnejad [15,16]. Patients with incomplete data excluded; MDS-UPDRS, Movement Disorder Society–Unified Parkinson’s Disease Rating Scale; MoCA, Montreal Cognitive Assessment; H&Y, Hoehn & Yahr; SAA, seed amplification assay. Indeterminate patients (n = 131) excluded.

**Table 2:** Genetic variant carrier frequencies by clinical motor subtype (TD vs. PIGD).

Genetic Variant	PIGD (N = 270), n (%)	TD (N = 739), n (%)	Test	*p*-Value	q-Value	Cramér’s V
*LRRK2*—All	19/270 (7.0)	25/739 (3.4)	χ^2^	0.024	0.095	0.07
*GBA*—All	8/270 (3.0)	23/739 (3.1)	χ^2^	1.0	1.0	0.00
*SNCA*—All	2/270 (0.7)	0/739 (0.0)	Fisher	0.074	0.236	0.05
*PRKN*	3/270 (1.1)	12/739 (1.6)	Fisher	0.771	0.949	0.01
*PINK1*	0/270 (0.0)	0/739 (0.0)	—	—	—	—
*PARK7*	0/270 (0.0)	0/739 (0.0)	—	—	—	—
*VPS35*	0/270 (0.0)	0/739 (0.0)	—	—	—	—
Any variant	32/270 (11.9)	59/739 (8.0)	χ^2^	0.096	0.288	0.05
*APOE*—ε4	61/269 (22.7)	179/731 (24.5)	χ^2^	0.539	0.762	0.02
*APOE*—ε2	41/269 (15.2)	114/731 (15.6)	χ^2^	0.902	1.0	0.00
*LRRK2*—*G2019S*	17/269 (6.3)	22/739 (3.0)	χ^2^	0.031	0.113	0.07
*LRRK2*—*R1441*	1/269 (0.4)	3/739 (0.4)	Fisher	1.0	1.0	0.00
*GBA*—*N409S*	6/211 (2.8)	16/616 (2.6)	χ^2^	1.0	1.0	0.00
*GBA*—Severe	2/211 (0.9)	7/616 (1.1)	Fisher	1.0	1.0	0.00
*SNCA*—*A53T*	2/211 (0.9)	0/616 (0.0)	Fisher	0.074	0.236	0.05

Carrier frequencies reported as n/N (%). χ^2^, Pearson’s chi-square test; Fisher, Fisher’s exact test. q-values are Benjamini–Hochberg FDR-adjusted *p*-values across all 48 comparisons. Cramér’s V quantifies effect size. — indicates not estimable (zero carriers in both groups).

**Table 3:** Genetic variant carrier frequencies by SAA status (SAA+ vs. SAA−).

Genetic Variant	SAA+ (N = 1069), n (%)	SAA− (N = 151), n (%)	Test	*p*-Value	q-Value	Cramér’s V
*LRRK2*—All	109/1069 (10.2)	56/151 (37.1)	χ^2^	3.7 × 10^−19^	< 0.001	0.25
*GBA*—All	80/1069 (7.5)	7/151 (4.6)	χ^2^	0.279	0.514	0.03
*SNCA*—All	12/1069 (1.1)	0/151 (0.0)	Fisher	0.381	0.614	0.02
*PRKN*	11/1069 (1.0)	3/151 (2.0)	Fisher	0.400	0.620	0.02
*PINK1*	0/1069 (0.0)	0/151 (0.0)	—	—	—	—
*PARK7*	0/1069 (0.0)	0/151 (0.0)	—	—	—	—
*VPS35*	0/1069 (0.0)	0/151 (0.0)	—	—	—	—
Any variant	205/1069 (19.2)	65/151 (43.0)	χ^2^	6.4 × 10^−11^	< 0.001	0.18
*APOE*—ε4	254/1059 (24.0)	37/151 (24.5)	χ^2^	0.887	1.0	0.00
*APOE*—ε2	151/1059 (14.3)	27/151 (17.9)	χ^2^	0.257	0.494	0.03
*LRRK2*—*G2019S*	103/1069 (9.6)	43/151 (28.5)	χ^2^	4.9 × 10^−11^	< 0.001	0.18
*LRRK2*—*R1441*	5/1069 (0.5)	12/151 (7.9)	χ^2^	2.7 × 10^−12^	< 0.001	0.20
*GBA*—*N409S*	66/916 (7.2)	6/124 (4.8)	χ^2^	0.384	0.614	0.02
*GBA*—Severe	14/916 (1.5)	1/124 (0.8)	Fisher	1.0	1.0	0.01
*SNCA*—*A53T*	12/916 (1.3)	0/124 (0.0)	Fisher	0.381	0.614	0.02

Carrier frequencies reported as n/N (%). χ^2^, Pearson’s chi-square test; Fisher, Fisher’s exact test. q-values are Benjamini–Hochberg FDR-adjusted *p*-values across all 48 comparisons. Cramér’s V quantifies effect size. — indicates not estimable.

**Table 4:** Genetic variant carrier frequencies by pathological subtype (body-first vs. brain-first).

Genetic Variant	Body-First (N = 302), n (%)	Brain-First (N = 879), n (%)	Test	*p*-Value	q-Value	Cramér’s V
*LRRK2*—All	24/302 (7.9)	132/879 (15.0)	χ^2^	0.002	0.013	0.08
*GBA*—All	37/302 (12.3)	59/879 (6.7)	χ^2^	0.004	0.021	0.08
*SNCA*—All	9/302 (3.0)	13/879 (1.5)	χ^2^	0.164	0.394	0.04
*PRKN*	4/302 (1.3)	10/879 (1.1)	Fisher	0.764	0.949	0.00
*PINK1*	0/302 (0.0)	0/879 (0.0)	—	—	—	—
*PARK7*	0/302 (0.0)	0/879 (0.0)	—	—	—	—
*VPS35*	0/302 (0.0)	0/879 (0.0)	—	—	—	—
Any variant	72/302 (23.8)	210/879 (23.9)	χ^2^	0.978	1.0	0.00
*APOE*—ε4	81/301 (26.9)	199/872 (22.8)	χ^2^	0.200	0.436	0.04
*APOE*—ε2	43/301 (14.3)	131/872 (15.0)	χ^2^	0.803	0.963	0.01
*LRRK2*—*G2019S*	21/302 (7.0)	117/878 (13.3)	χ^2^	0.004	0.020	0.08
*LRRK2*—*R1441*	3/302 (1.0)	14/878 (1.6)	Fisher	0.583	0.799	0.01
*GBA*—*N409S*	30/251 (12.0)	49/741 (6.6)	χ^2^	0.015	0.067	0.07
*GBA*—Severe	7/251 (2.8)	10/741 (1.3)	χ^2^	0.237	0.494	0.03
*SNCA*—*A53T*	9/251 (3.6)	13/741 (1.8)	χ^2^	0.164	0.394	0.04

Carrier frequencies reported as n/N (%). χ^2^, Pearson’s chi-square test; Fisher, Fisher’s exact test. q-values are Benjamini–Hochberg FDR-adjusted *p*-values across all 48 comparisons. Cramér’s V quantifies effect size. — indicates not estimable.

**Table 5:** Genetic variant carrier frequencies by data-driven subtype (DM vs. IM vs. MMP).

Genetic Variant	DM (N = 279), n (%)	IM (N = 447), n (%)	MMP (N = 407), n (%)	Test	*p*-value	q-value	Cramér’s V
*LRRK2*—All	42/279 (15.1)	57/447 (12.8)	37/407 (9.1)	χ^2^	0.108	0.305	0.06
*GBA*—All	39/279 (14.0)	28/447 (6.3)	24/407 (5.9)	χ^2^	3.4 × 10^−4^	0.003	0.11
*SNCA*—All	8/279 (2.9)	7/447 (1.6)	9/407 (2.2)	χ^2^	0.497	0.723	0.03
*PRKN*	4/279 (1.4)	4/447 (0.9)	6/407 (1.5)	χ^2^	0.672	0.895	0.03
*PINK1*	0/279 (0.0)	0/447 (0.0)	0/407 (0.0)	—	—	—	—
*PARK7*	0/279 (0.0)	0/447 (0.0)	0/407 (0.0)	—	—	—	—
*VPS35*	0/279 (0.0)	0/447 (0.0)	0/407 (0.0)	—	—	—	—
Any variant	90/279 (32.3)	95/447 (21.3)	74/407 (18.2)	χ^2^	3.7 × 10^−4^	0.003	0.11
*APOE*—ε4	58/279 (20.8)	119/443 (26.9)	93/403 (23.1)	χ^2^	0.182	0.417	0.05
*APOE*—ε2	41/279 (14.7)	69/443 (15.6)	52/403 (12.9)	χ^2^	0.718	0.932	0.02
*LRRK2*—*G2019S*	40/277 (14.4)	51/447 (11.4)	35/407 (8.6)	χ^2^	0.122	0.325	0.06
*LRRK2*—*R1441*	1/277 (0.4)	6/447 (1.3)	2/407 (0.5)	χ^2^	0.255	0.494	0.05
*GBA*—*N409S*	32/235 (13.6)	23/371 (6.2)	16/345 (4.6)	χ^2^	3.6 × 10^−4^	0.003	0.11
*GBA*—Severe	7/235 (3.0)	5/371 (1.3)	8/345 (2.3)	χ^2^	0.356	0.614	0.04
*SNCA*—*A53T*	8/236 (3.4)	7/371 (1.9)	9/345 (2.6)	χ^2^	0.497	0.723	0.03

Carrier frequencies reported as n/N (%). DM, diffuse malignant; IM, intermediate; MMP, mild-motor predominant. χ^2^, Pearson’s chi-square test; Fisher, Fisher’s exact test. q-values are Benjamini–Hochberg FDR-adjusted *p*-values across all 48 comparisons. Cramér’s V quantifies effect size. — indicates not estimable.

## Data Availability

PPMI data are available here upon request: https://www.ppmi-info.org/.

## Supplementary Materials

The following supporting information can be downloaded at: https://www.mdpi.com/article/10.3390/genes17040449/s1, Figure S1. *APOE* Genotype Distribution Across Subtyping Frameworks. Distribution of *APOE* genotypes (ε2/ε2, ε2/ε3, ε2/ε4, ε3/ε3, ε3/ε4, ε4/ε4) across (a) Clinical motor subtypes (TD/PIGD), (b) SAA status (SAA+/SAA−), (c) Pathological subtypes (Brain-first/Body-first), and (d) Data-driven subtypes (DM/IM/MMP). No statistically significant differences were observed in any framework (all *p* > 0.16). Figure S2. Cross-Scheme Agreement Between Subtyping Frameworks. Cramér’s V heatmap showing pairwise concordance between the four subtyping frameworks. The highest agreement was between Data-driven and Pathological and Data-driven and Clinical classifications, indicating that the four frameworks capture largely non-overlapping aspects of PD heterogeneity. Figure S3. Adjusted Odds Ratios from Multivariable Logistic Regression (Exploratory Analysis). Table–forest hybrid showing adjusted ORs (95% Wald CI) for five predictors across four subtyping comparisons. Colored, bold entries indicate *p* < 0.05. Notable associations: *LRRK2* protective for SAA+ (OR = 0.22), male sex associated with body-first (OR = 2.14), age with body-first (OR = 1.37) and DM (OR = 1.63). Log scale. These regression models should be interpreted as exploratory given the reduced sample sizes (N = 560–600) from complete-case analysis; the FDR-corrected univariate results (Table 2, Table 3, Table 4 and Table 5, main text) provide the primary statistical framework. OR, odds ratio; CI, confidence interval; *LRRK2*, leucine-rich repeat kinase 2; *GBA1*, glucocerebrosidase; *APOE*, apolipoprotein E; SAA, seed amplification assay; DM, diffuse malignant.

## Abbreviations

The following abbreviations are used in this manuscript:

PD	Parkinson’s disease
TD	Tremor-dominant
PIGD	Postural instability/gait difficulty
SAA	Seed amplification assay
DM	Diffuse malignant
MMP	Mild-motor predominant
IM	Intermediate
PPMI	Parkinson’s Progression Markers Initiative
*LRRK2*	Leucine-rich repeat kinase 2
*GBA1*	Glucocerebrosidase
*SNCA*	Synuclein alpha
*APOE*	Apolipoprotein E
MDS-UPDRS	Movement Disorder Society–Unified Parkinson’s Disease Rating Scale
MoCA	Montreal Cognitive Assessment
RBD	Rapid eye movement sleep behavior disorder
RBDSQ	RBD Screening Questionnaire
CSF	Cerebrospinal fluid
CLIA	Clinical Laboratory Improvement Amendments
OR	Odds ratio
CI	Confidence interval
FDR	False discovery rate
AIC	Akaike information criterion
IQR	Interquartile range
SD	Standard deviation
SCOPA-AUT	Scales for Outcomes in Parkinson’s Disease–Autonomic
