# Supplementary material for: Genetic Associations of Parkinson’s Disease Clinical, Pathological, and Data-Driven Subtypes
Source: Genes (Basel). 2026 Apr 13;17(4):449. doi: 10.3390/genes17040449 (PMC13116718; doi:10.3390/genes17040449)
Supplement: Supplementary file 1 [file genes-17-00449-s001.zip › genes-4218140-supplementary.pdf]

# Supplementary Materials

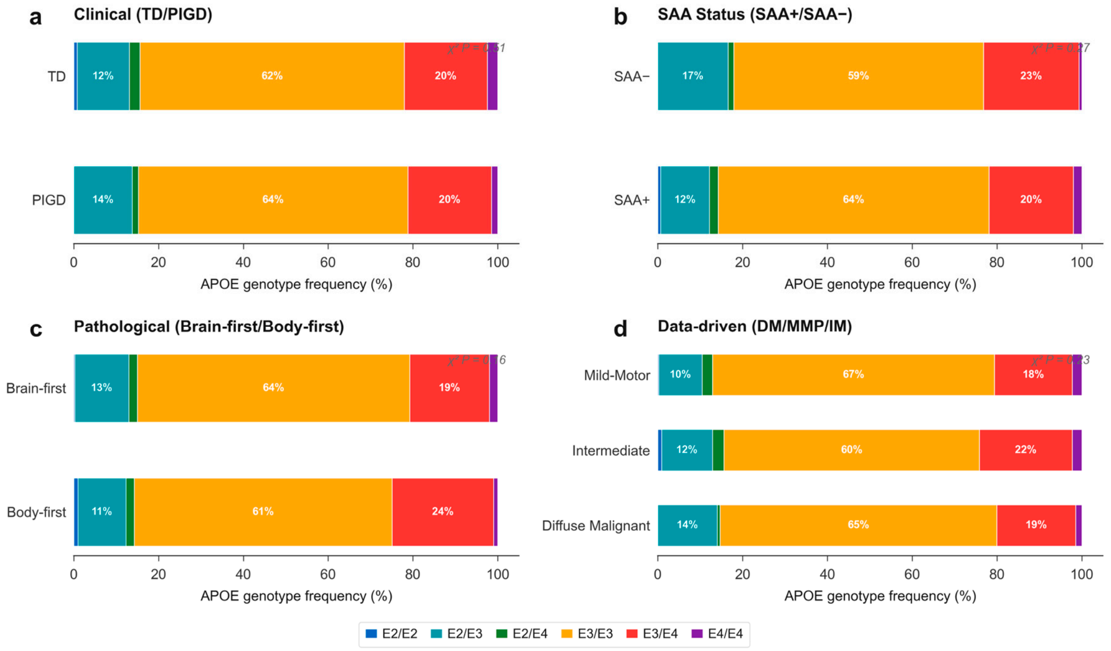


**Supplementary Figure S1.** APOE Genotype Distribution Across Subtyping Frameworks. Distribution of APOE genotypes (ε2/ε2, ε2/ε3, ε2/ε4, ε3/ε3, ε3/ε4, ε4/ε4) across (a) Clinical motor subtypes (TD/PIGD), (b) SAA status (SAA+/SAA−), (c) Pathological subtypes (Brain-first/Body-first), and (d) Data-driven subtypes (DM/IM/MMP). No statistically significant differences were observed in any framework (all P > 0.16).

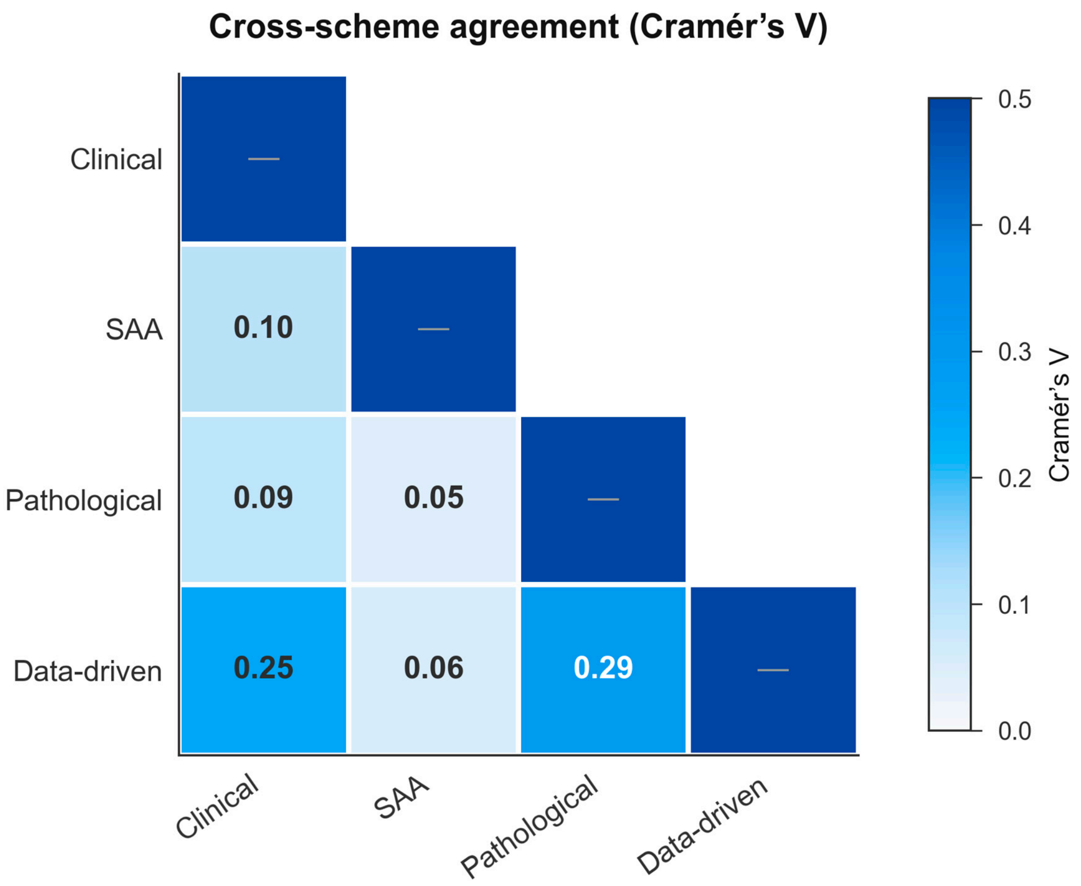


**Supplementary Figure S2.** Cross-Scheme Agreement Between Subtyping Frameworks. Cramér's V heatmap showing pairwise concordance between the four subtyping frameworks. The highest agreement was between Data-driven and Pathological (V = 0.29) and Data-driven and Clinical (V = 0.25) classifications, indicating that the four frameworks capture largely non-overlapping aspects of PD heterogeneity.

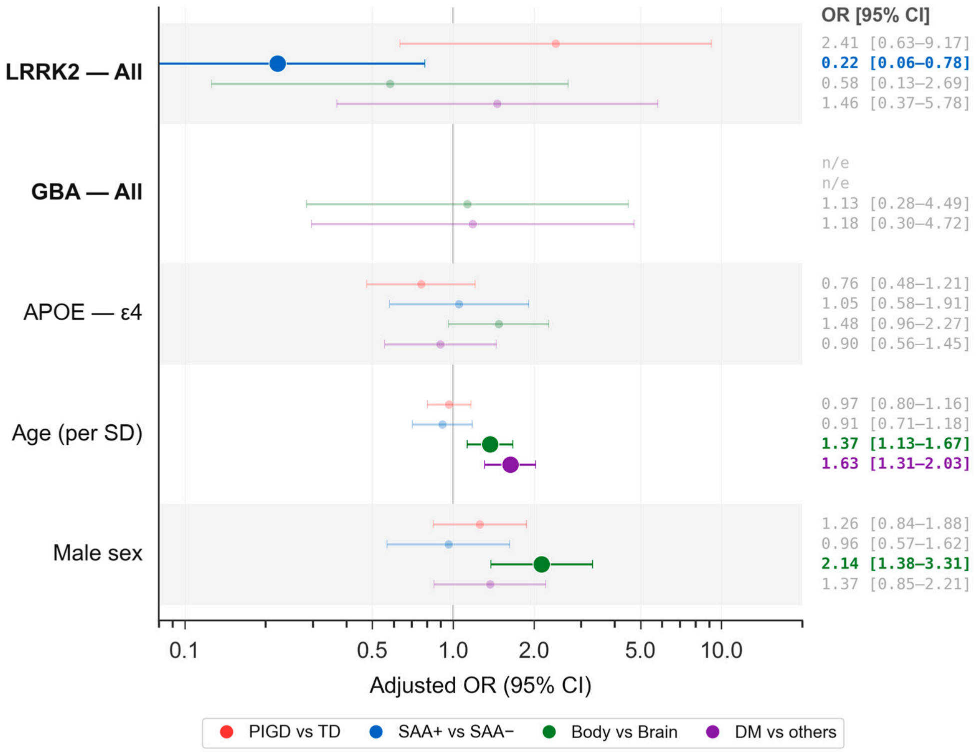


**Supplementary Figure S3.** Adjusted Odds Ratios from Multivariable Logistic Regression (Exploratory Analysis). Table–forest hybrid showing adjusted ORs (95% Wald CI) for five predictors across four subtyping comparisons. Colored, bold entries indicate P < 0.05. Notable associations: *LRRK2* protective for SAA+ (OR = 0.22), male sex associated with body-first (OR = 2.14), age with body-first (OR = 1.37) and DM (OR = 1.63). Log scale. These regression models should be interpreted as exploratory given the reduced sample sizes (N = 560–600) from complete-case analysis; the FDR-corrected univariate results (Tables 2–5, main text) provide the primary statistical framework. OR, odds ratio; CI, confidence interval; *LRRK2*, leucine-rich repeat kinase 2; *GBA1*, glucocerebrosidase; *APOE*, apolipoprotein E; SAA, seed amplification assay; DM, diffuse malignant.
